# IgG Responses to *Anopheles gambiae* Salivary Antigen gSG6 Detect Variation in Exposure to Malaria Vectors and Disease Risk

**DOI:** 10.1371/journal.pone.0040170

**Published:** 2012-06-29

**Authors:** Will Stone, Teun Bousema, Sophie Jones, Samwel Gesase, Rhamadhan Hashim, Roly Gosling, Ilona Carneiro, Daniel Chandramohan, Thor Theander, Raffaele Ronca, David Modiano, Bruno Arcà, Chris Drakeley

**Affiliations:** 1 Department of Immunity and Infection, London School of Hygiene and Tropical Medicine, London, United Kingdom; 2 Department of Medical Microbiology, Radboud University Nijmegen Medical Centre, Nijmegen, The Netherlands; 3 National Institute for Medical Research, Tanga, Tanzania; 4 Global Health Group, University of California San Francisco (UCSF), San Francisco, California, United States of America; 5 Centre for Medical Parasitology at Department of International Health, Immunology and Microbiology, University of Copenhagen and Department of Infectious Diseases, Copenhagen University Hospital, Copenhagen, Denmark; 6 Department of Structural and Functional Biology, University “Federico II”, Naples, Italy; 7 Parasitology Section, Department of Public Health and Infectious Diseases, University “La Sapienza”, Rome, Italy; Universidade Federal de Minas Gerais, Brazil

## Abstract

Assessment of exposure to malaria vectors is important to our understanding of spatial and temporal variations in disease transmission and facilitates the targeting and evaluation of control efforts. Recently, an immunogenic *Anopheles gambiae* salivary protein (gSG6) was identified and proposed as the basis of an immuno-assay determining exposure to Afrotropical malaria vectors. In the present study, IgG responses to gSG6 and 6 malaria antigens (CSP, AMA-1, MSP-1, MSP-3, GLURP R1, and GLURP R2) were compared to *Anopheles* exposure and malaria incidence in a cohort of children from Korogwe district, Tanzania, an area of moderate and heterogeneous malaria transmission. Anti-gSG6 responses above the threshold for seropositivity were detected in 15% (96/636) of the children, and were positively associated with geographical variations in *Anopheles* exposure (OR 1.25, CI 1.01–1.54, p = 0.04). Additionally, IgG responses to gSG6 in individual children showed a strong positive association with household level mosquito exposure. IgG levels for all antigens except AMA-1 were associated with the frequency of malaria episodes following sampling. gSG6 seropositivity was strongly positively associated with subsequent malaria incidence (test for trend p = 0.004), comparable to malaria antigens MSP-1 and GLURP R2. Our results show that the gSG6 assay is sensitive to micro-epidemiological variations in exposure to *Anopheles* mosquitoes, and provides a correlate of malaria risk that is unrelated to immune protection. While the technique requires further evaluation in a range of malaria endemic settings, our findings suggest that the gSG6 assay may have a role in the evaluation and planning of targeted and preventative anti-malaria interventions.

## Introduction

Heterogeneity in malaria exposure is present at all levels of endemicity [1] but is most readily observed in areas of low transmission and following periods of extensive control [1]–[3]. Recent evidence of decreasing malaria incidence [2], [4], has fuelled calls for malaria elimination from the world’s public health, political and philanthropic authorities [5], [6]. As a result the interest in malaria heterogeneity and its potential effect on malaria control has increased [2], [3], [7]. Hotspots of higher malaria transmission are likely to hamper malaria elimination efforts, as residual foci of persistent malaria infection may seed transmission to the wider community [8]–[10].

Although not all factors that affect malaria heterogeneity are fully understood, variation in the exposure to malaria vectors is likely to be of key importance [3], [11]–[13]. In sub-Saharan Africa, the transmission of *Plasmodium falciparum* is maintained by three key mosquito species; *Anopheles gambiae*, *An. arabiensis* and *An. funestus* [14]. Mosquito exposure is typically assessed as a component of the entomological inoculation rate (EIR), which is defined as the number of infectious *Anopheles* bites per person per unit time (ib/p/yr) [15], [16]. Despite its value in malaria research, a direct assessment of EIR to determine small-scale variation in malaria exposure is operationally unattractive at low levels of transmission (EIR<10 ib/p/yr) [17]–[19]. The development of accurate and sensitive tools for identifying micro-epidemiological variations in vector exposure and malaria risk is important in assessing the efficiency of control efforts and focusing interventions to those areas or populations that are most affected by malaria. Serological assessments of malaria exposure are receiving increasing interest in this respect and have been used for quantifying malaria transmission intensity [20] and its temporal [21] and spatial variation [11], [22], [23]. Recently, serological markers of malaria exposure were also used to quantify heterogeneity in the efficacy of malaria interventions [24]. Recombinant malaria blood stage antigens have been most widely used for these purposes [25], while responses to the infective sporozoite specific circum-sporozoite protein (CSP) are currently viewed as the best available serological tool to detect exposure to infectious mosquito bites [18], [26]–[28]. A similar tool to identify spatial patterns of cumulative exposure to *Anopheles* biting could be integral to the detection of malaria hotspots and play a role in forecasting the risk of malaria epidemics or the dynamics of malaria resurgence in areas where parasite carriage in human populations has decreased but exposure to malaria vectors persists [29].

Our understanding of the human immune response to mosquito saliva has until recently been largely restricted to culicine mosquitoes and the clinical consequences of allergy [30]–[32]. Humoral responses to the saliva of various disease vectors have been exploited epidemiologically, revealing significant correlation with disease seropositivity and vector exposure. Such assays have now been described for *Ixodes* ticks [33], [34], triatomine bugs [35], *Glossina* tsetse flies [36] and *Lutzomyia* and *Phlebotomus* sand flies [37], [38]. Recently, transcriptome analysis of the salivary glands of *An. gambiae* females identified over 70 putative secreted salivary proteins [39]–[41]. A small (∼10 kb) immunogenic protein, gambiae salivary gland protein 6 (gSG6), that is well conserved in the three major Afrotropical malaria vectors (*An. gambiae, An. arabiensis* and *An. funestus*) and restricted to anopheline mosquitoes [42], has been identified as a suitable candidate for a bioassay of *Anopheles* exposure [43], [44]. Antibody responses to a gSG6 peptide (gSG6-P1) described *Anopheles* exposure in areas of low vector density [45] and in response to vector control programs [46] with some success, and were recently shown to reflect *Anopheles* heterogeneity at the district level in Dakar, Senegal [47]. Recombinant full length gSG6 has also shown strong immunogenicity among rural populations in Burkina Faso, which appears to be sufficiently short lived to correlate with seasonal changes in *Anopheles* abundance [43], [48].The relationship between malaria case incidence and anti-gSG6 response has not been studied, despite early indications that humoral responses to *Anopheles* whole saliva were positively associated with malaria infection [49].

Using a subset of samples collected during a large study of intermittent presumptive treatment among infants (IPTi) [50], along with entomological data from an intensive survey in the same area [11], we present the first evaluation of IgG antibody responses to the recombinant gSG6 salivary antigen for describing spatial heterogeneity in vector exposure between and within geographically defined subvillages in an area of moderate and heterogeneous malaria exposure in northern Tanzania. At the individual level, we determine the association of gSG6 reactivity with household *Anopheles* exposure and subsequent malaria incidence. In addition, we determined reactivity against a selection of malaria antigens that have been more commonly used in epidemiological studies, namely CSP and four blood stage proteins, AMA-1, MSP-1, MSP-3, and glutamate-rich protein (GLURP).

## Methods

### Ethics Statement

Witnessed written consent was provided by the caregivers of all children involved in serological sampling, and by heads of households for participation in the entomological survey. Ethical approval was granted by the review board of the National Institute for Medical Research of Tanzania, and the London School of Hygiene and Tropical Medicine ethics committee.

### Study Area and Subjects

Plasma samples were collected from children recruited over 18 months as part of a longer-term study (2004–2008) carried out in the district of Korogwe, Northern Tanzania, an area of moderate malaria endemicity. Korogwe district is situated ∼600 m above sea level, and has a seasonal pattern of rainfall (800–1400 mm/year) [50]. Malaria transmission in the Korogwe region has declined in recent years [51], such that an EIR of 1–14 ib/p/yr was estimated in 2007 [21]. The original study investigated the relative impacts of different drug regimens for intermittent presumptive treatment (IPTi) among a total of 1280 infants [50].

### Entomological Data Collection

In the final year of the IPTi study a randomly selected subset of 600 children were enrolled in a detailed entomological survey, aiming to describe spatial patterns of malaria incidence in relation to mosquito exposure [11]. In the room of each selected child, mosquitoes were sampled with miniature CDC light traps (Model 512; John W. Hock Company, Gainesville, Florida) for one night at the end of the wet season (May), again at the beginning (July) and finally the end (September) of the dry season in 2008. Mosquito exposure at the household level was highly correlated between all surveys (correlation coefficient: May/July = 0.462, May/September = 0.497, July/September = 0.444; p<0.0001). Mosquito data from first of the three sampling points, during the peak transmission season when *Anopheles* abundance was highest, was therefore deemed adequate in displaying variation in exposure. Of the total *Anopheles* females caught during sampling, *An. gambiae s.l.* made up 80.3%, *An. funestus* 18.6% and other anophelines 1%.

### Clinical Data and Plasma Samples

Malaria incidence was assessed by passive monitoring for signs of illness throughout the 22 months following recruitment, during which time free access to clinical treatment was provided [50]. The average age at recruitment was 9.4 weeks (range 8–17 weeks) and infants were recruited at different times of the year, i.e. at different time-points in the transmission season. Plasma samples used in the current study were taken at 9 months of age when infants were presented at clinics as part of the Expanded Program on Immunisation (EPI). Blood samples were collected by finger prick and after plasma separation samples were stored at −20°C until processing. In our analyses, we included malaria incidence in the period between serum collection at 9 months of age and the end of follow-up. This gave an effective follow up period of approximately 15 months and ensured that the follow-up period included one or more peak malaria transmission seasons for each child. The current analyses are an ancillary study and many of the blood samples had been used previously for other IPTi specific investigations. As a result of this non-systematic exhaustion of samples, sera were available for a subset of 636/1280 children for gSG6 ELISA; 247/636 children from this subset were involved in the household level entomological survey.

### gSG6 ELISA

ELISA was performed as previously described with few modifications [43], [48]. Briefly, Maxisorp 96-well plates (Nunc M9410) were coated with gSG6 at 5 ug/ml. Test and negative control serum were analysed in duplicate at 1∶100 in phosphate buffered saline with 0.05% Tween 20 (PBST)/1% skimmed milk powder (Marvel, UK). On every plate blank wells (PBST/Marvel) were included to correct sample ODs for background antibody reactivity, and positive control sera (1∶40 in PBST/Marvel) were analysed to allow standardisation of OD values for day-to-day and inter-plate variation. Positive control sera was provided, with consent, by an employee of the London School of Hygiene and Tropical medicine who was exposed weekly to the bites of approximately 50–100 laboratory bred *An. gambiae s.s* (Kisumu strain) during colony feeding.

Sera from 39 Europeans with no recent history of travel to malaria endemic countries were used as negative controls for calculation of IgG seroprevalence. Cut off for seropositivity among samples was determined as the mean OD of the unexposed sera plus 3 standard deviations.

### *P. falciparum* ELISA and Luminex Assays

For this analysis, IgG antibody responses were chosen in preference to IgM for their high antigen specificity. IgG antibody responses against CSP (Gennova, 0.009 µg/ml), AMA-1 (BPRC, 0.3 µg/ml) and MSP-1_19_ (CTK Biotech, 0.2 µg/ml) were detected as previously described [20], [27]. Test sera were analysed in duplicate at 1∶200 (CSP), 1∶1000 (MSP-1_19_) or 1∶2000 (AMA-1) in PBST/Marvel. Blank wells, positive control sera from a hyper-endemic region in the Gambia [20], and a serial dilution of pooled hyper-immune sera were included in duplicate on each plate to correct for non-specific reactivity and allow standardisation of inter-plate variation. Seroprevalence of IgG antibodies to these non-salivary antigens was calculated using a mixture model as described previously [20], [52].

Recombinant proteins corresponding to the R1, R2 (Central repeat and C-terminal repeat regions of GLURP), and the C-terminal region of MSP-3 [53] were covalently coupled to carboxylated luminex microspheres according to the manufacturer’s protocol and tested as previously described [54]. Cut-off for positivity was calculated as the mean reactivity in malaria non-exposed European individuals plus 2 standard deviations.

### Data Analysis

**Figure 1:**
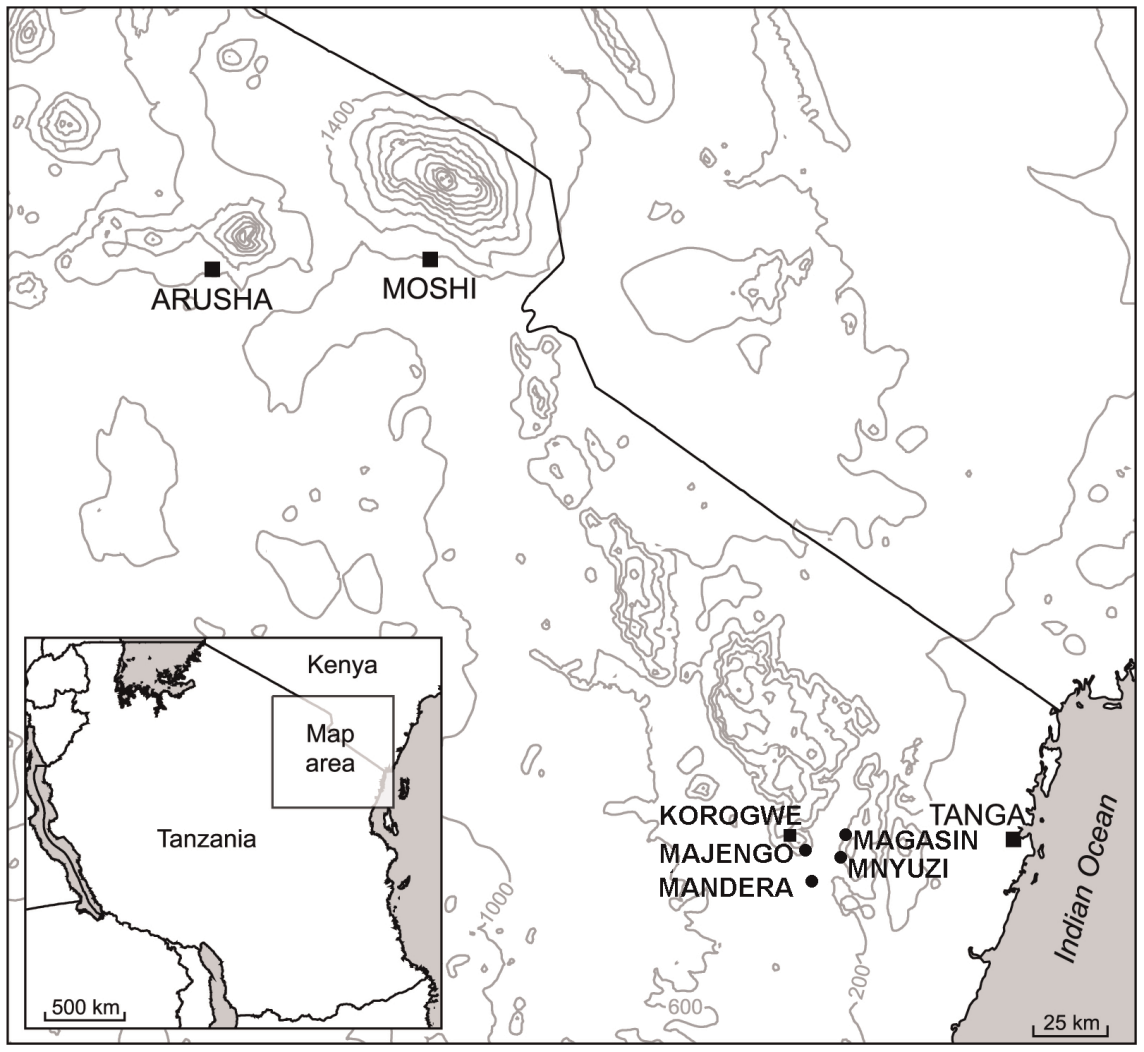
Map of Tanzania showing the north-eastern provinces, and the location of Korogwe district. Sampling in Korogwe district was conducted in 5 areas, which are marked on the map: Korogwe, Majengo, Magasin, Mnyuzi, and Mandera. Within these areas, our study population were resident in 15 subvillages. Korogwe consisted of the following subvillages: Kwasemangube (KS), Lwengera (LW) Msambazi (MS) and Masuguru (MU). Majengo consisted of the following subvillages: Kilole (KI), Majengo (MJ) and Manundu (MA). Magasin consisted of the following subvillages: Kwagunda (KW) and Maguga (MG)**.** Mnyuzi consisted of the following subvillages: Gereza (GE), Lusanga (LU), Mkwakwani (MK), Mnyuzi (MY) and Shambakapori (SH)**.** Mandera (MD) was an isolated subvillage.

**Figure 2:**
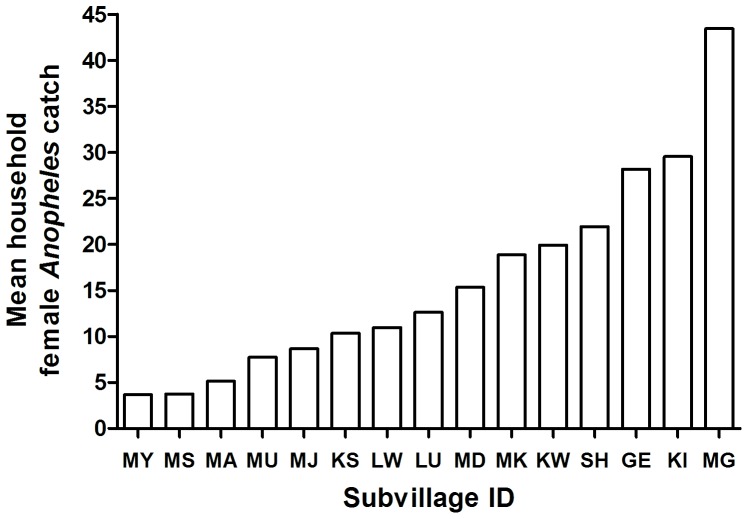
Mean household *Anopheles* female count during peak transmission (May) in different subvillages. Numbers of households sampled for each subvillage, in order of *Anopheles* exposure, were as follows: MY = 45, MS = 23, MA = 26, MU = 21, MJ = 29, KS = 24, LW = 65, LU = 61, MD = 45, MK = 14, KW = 99, SH = 13, GE = 47, KI = 30, MG = 91.

To examine the relationship between patterns of gSG6 reactivity and small scale spatial variation in *Anopheles* exposure, antibody responses were described at the level of subvillages, which are defined by their geographical location (*Figure 1*) [11]. The arithmetic mean mosquito exposure for each village was used for ranking villages from low to high mosquito exposure; this rank was related to antibody prevalence and mean log 10 adjusted antibody level per subvillage. This enabled analyses relating to geographic variations in *Anopheles* abundance for all individuals, irrespective of their involvement in the entomological survey (*Figure 2*).

For infants for whom both household mosquito data and plasma samples were available, it was possible to investigate associations between *Anopheles* exposure and antibody reactivity against salivary and malaria antigens at an individual level. For this purpose, households were analysed in quintiles of *Anopheles* exposure (*Table 1*).

**Table 1:** Households grouped into quintiles according to their relative exposure to *Anopheles* females during the wet season entomological survey (May).

		Female *Anopheles* per household	
Quintile	Households	Mean	Range
1	64	0	0
2	44	1.59	1–2
3	45	4.11	3–5
4	45	11.71	6–17
5	49	43.37	17–119

Statistical analysis was conducted in STATA (Version 10, STATA statistical software StataCorp) and GraphPad Prism (Version 5.0, GraphPad Software Inc., La Jolla, CA) software packages. IgG responses to salivary or malaria antigens between two independent groups were compared by Wilcoxon rank-sum tests (Mann-Whitney U test), with Bonferroni correction for multiple comparisons between subgroups. Comparisons of multiple groups were carried out by Kruskal-Wallis test. Seroprevalence comparisons were made using Chi-square test, with a test for trend in proportions. Correlations between IgG and malaria or entomological measures were made using Spearman correlation or with linear regression analysis after log10-transformation of OD data. IPTi treatment arm was included in our analyses as potential confounder. As a small number of sample ODs were lower than their ELISA plate blank value, some normalised ODs had negative values and an arbitrary positive value (+1) was therefore added to all ODs before transformation.

## Results

### Small Scale Spatial Variation in *Anopheles* Exposure and anti-gSG6 Responses

The recombinant gSG6 protein elicited significant anti-gSG6 IgG responses in children from Korogwe district (mean OD 0.109, maximum OD 2.014). European sera were used as negative controls for exposure to *Anopheles* mosquitoes, the responses of which were pooled to determine a cut-off for seroprevalence at OD 0.167 (*Table 2*). Mean OD among antibody negative children from Korogwe was 0.052, and ranged from 0.001–0.166 (standard deviation 0.040). IPTi treatment arm was not associated with gSG6 antibody prevalence (p = 0.23) or density (p = 0.38) and did not show any evident association with any of the other antigens tested, nor was it found to be a confounder in any of the associations presented below (data not shown).

**Table 2:** Seroprevalence and IgG antibody levels among seropositive children to *An. gambiae* gSG6, and *P. falciparum* CSP, AMA-1, MSP-1, MSP-3, GLURP R1 and GLURP R2.

	gSG6	CSP	AMA-1	MSP-1	MSP-3	GLURP R1	GLURP R2
**Antibody prevalence % (n/N)**	15 (96/636)	21 (121/575)	2 (9/540)	10 (52/540)	10 (54/566)	3 (16/566)	12 (67/566)
Median OD (IQR)*	0.290 (0.213–0.575)	0.464 (0.375–0.743)	0.087 (0.066–0.110)	0.210 (0.110–0.329)	−	−	−

**OD** optical density.

**IQR** inter-quartile range (25^th^ and 75^th^ percentiles).

**n/N** proportion of seropositive individuals/total sample size.

* seropositive individuals only.

**Figure 3:**
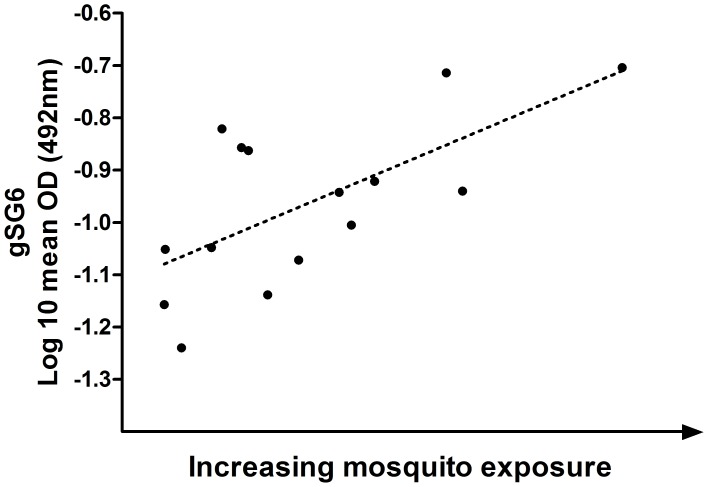
Mean anti-gSG6 IgG level per subvillage, plotted against increasing mosquito exposure per subvillage. Anti-gSG6 IgG levels are given as the log-10 adjusted mean anti-gSG6 OD per subvillage. Mosquito exposure is given as the ascending and sequential mean *Anopheles* female count for each of 15 subvillages (x-axis), as in *Figure 2*. The trend-line from the linear regression is shown as a dashed line (r^2^ = 0.436, p = 0.007).

When mean mosquito exposure was plotted against log 10 adjusted anti-gSG6 IgG level for each of the 15 subvillages, a significant positive association was observed between mean mosquito exposure and antibody reactivity (*Figure 3*). Similarly, despite significant variability in gSG6 response between subvillages, there was a significant positive association between mean mosquito exposure per subvillage and anti-gSG6 IgG seropositivity, wherein an average increased exposure of 10 mosquitoes was associated with a 25% increase in antibody positivity (odds ratio [OR] 1.25, CI 1.01–1.54, p = 0.04).

### Household Level Mosquito Exposure and anti-gSG6 Response

**Figure 4:**
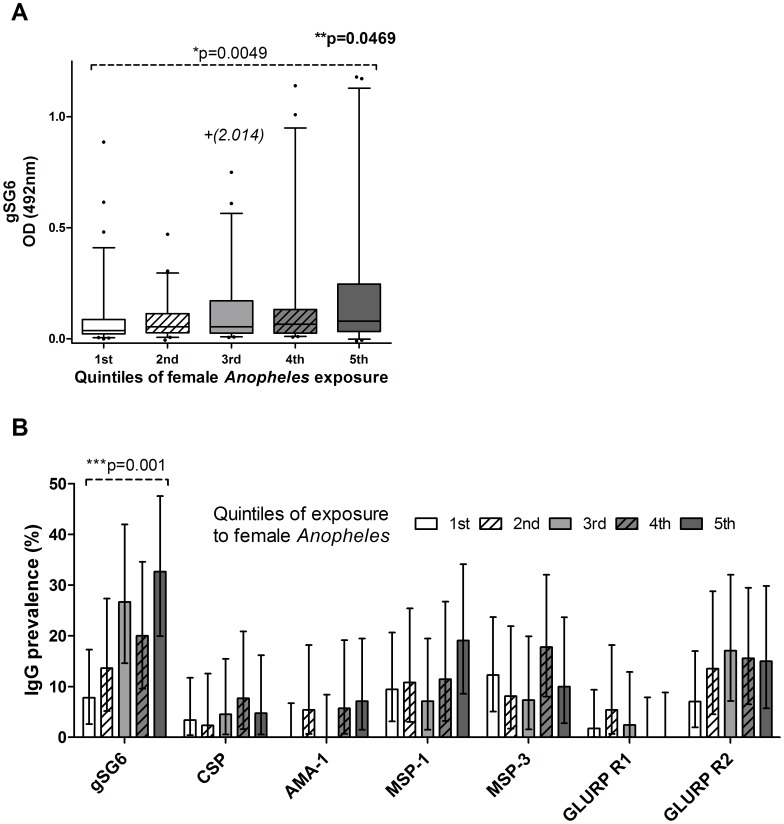
IgG responses to gSG6 and *P. falciparum* antigens, grouped into quintiles of household *Anopheles* exposure. **A.** Box plots showing anti-gSG6 IgG level between groups sorted according to *Anopheles* exposure in quintiles. Boxes show the median and 25^th^/75^th^ percentiles, whiskers show the 5^th^/95^th^ percentiles, and outliers are represented by dots. Where outliers were excluded from the graph but not analysis they are marked with a + and included in parentheses. P values for pairwise comparisons were determined by Mann-Whitney test with Bonferroni correction (*), and for all groups by Kruskal-Wallis test (**). **B.** Seroprevalence of anti-gSG6 and anti-*P. falciparum* IgG antibodies plotted against *Anopheles* exposure in quintiles. Error bars indicate 95% confidence intervals (CI). P values were determined by a test for trend in proportions (***).

Information on household-level mosquito exposure was available for the households of 247 children. At the level of individual households, exposure to *Anopheles* females showed a significant positive correlation with anti-gSG6 IgG level (correlation coefficient 0.188, p = 0.003) but not with levels of anti-CSP IgG (correlation coefficient 0.036, p = 0.59). When households were grouped into quintiles according to their relative exposure to *Anopheles* (*Table 1*), there was a statistically significant positive association between *Anopheles* exposure in quintiles and anti-gSG6 IgG levels (p = 0.001) and prevalence (test for trend in proportions, p = 0.001) (*Figure 4*). There was no evident association between individual *Anopheles* exposure in quintiles and individual CSP antibody level (p = 0.544) or prevalence (test for trend in proportions p = 0.422). Similarly, no significant associations were observed between *Anopheles* exposure in quintiles and individual responses to any blood stage antigen, save MSP-3 for which there was a significant positive association with antibody level (p = 0.017).

### Malaria Incidence and anti-gSG6 and Anti-malaria Responses

**Figure 5:**
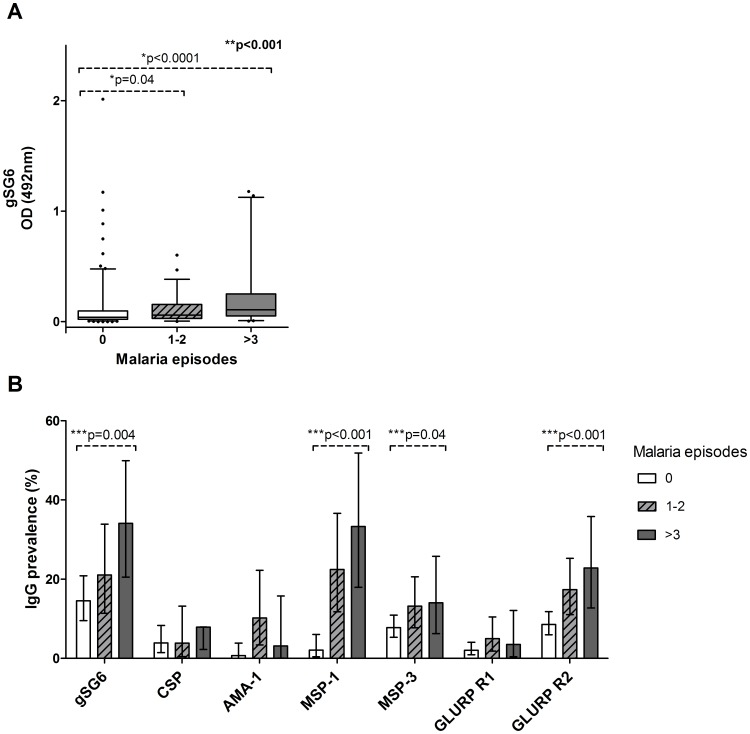
IgG responses to gSG6 and *P. falciparum* antigens, plotted against malaria incidence after serum collection. Malaria incidence is grouped into 0 episodes, 1–2 episodes or >3 episodes. **A.** Box plots showing anti-gSG6 IgG level between groups sorted according to malaria incidence subsequent to serological sampling. Boxes, whiskers and P values are as in *Figure 4*. n = 269. **B.** Seroprevalence of anti-gSG6 and anti-*P. falciparum* IgG antibodies plotted against grouped malaria incidence. Sample sizes vary by antigen according to the available serological methodology; CSP n = 246, AMA-1 n = 227, MSP-1 n = 227, MSP-3 n = 566, GLURP R1 n = 566, GLURP R2 n = 566. P values were determined by a test for trend in proportions (***). Error bars denote 95% CI.

Antibody levels were positively associated with the frequency of malaria episodes recorded after serum collection for all antigens except AMA-1 (gSG6 correlation coefficient 0.240, p<0.0001 (*Figure 5A*); CSP correlation coefficient 0.183, p = 0.004; MSP -1 correlation coefficient 0.256, p<0.0001; MSP-3 correlation coefficient 0.141, p = 0.0008; GLURP R1 correlation coefficient 0.126, p = 0.003; GLURP R2 correlation coefficient 0.101, p = 0.017 [data not shown]). The prevalence of IgG responses varied significantly with grouped malaria incidence for gSG6 (p<0.0001), AMA-1 (p = 0.004), MSP-1 (p<0.0001) and GLURP R2 (p = <0.001). No significant variation in seroprevalence of antibodies to CSP, MSP-3 and GLURP R1 was present between groups of malaria incidence (*Figure 5B*). A strong positive association was observed between grouped malaria incidence and the prevalence of antibody responses against gSG6, MSP-1 and GLURP R2, while this relationship was present but only marginally significant for MSP-3 (*Figure 5B*).

## Discussion

In the present study we show that the antibody responses of young children to the recombinant *An. gambiae* salivary protein, gSG6, reflect small scale spatial variation in malaria transmission, and are strongly associated with malaria risk in an area of moderate transmission intensity in northern Tanzania where *An. gambiae* and *An. funestus* are the main malaria vectors.

Reactivity to both the peptide and recombinant forms of the anopheline gSG6 protein has previously been associated with seasonal or regional patterns in mosquito exposure [45]–[48], [55]. The current study is the first to describe antibody responses to the recombinant gSG6 protein in relation to village of residence, and individual level mosquito exposure and malaria incidence. For this, we utilised a detailed entomological dataset from Korogwe district, Tanzania, that revealed significant heterogeneity in *Anopheles* abundance between and within villages [11]. Despite generally low reactivity among our infant study population, anti-gSG6 IgG level and prevalence effectively described varying levels of exposure to *Anopheles* between subvillages, corroborating recent findings from Senegal where gSG6-P1 responses reflected spatial variation in *Anopheles* exposure between districts in urban Dakar [47]. The first studies to assess IgG responses to recombinant gSG6 were carried out in two rural villages in Burkina Faso, and revealed >50% seroprevalence in children during the peak transmission season [48]. The lower responses observed in this study confirm the lower transmission intensity in the current study area.

At the level of subvillages, anti-gSG6 antibody responses closely followed patterns in malaria incidence and community-level antibody responses to malaria-specific antigens AMA-1 and MSP-1_19_ [11]. This broad agreement in estimates of malaria incidence and *Anopheles* and malaria-specific antibody responses at subvillage level is unsurprising [45], [48], [49], [55]. Patterns may diverge when assessed at an individual level, as *Anopheles* abundance and biting behaviour may be unevenly distributed between households [12], [21], [56] and intense mosquito exposure may not necessarily mean a high malaria exposure if anophelines are not infected. This commonly happens at the start of the wet season when mosquitoes have just emerged and are unlikely to have completed a sporogonic cycle [57], but mosquito sporozoite rates may also show spatial variation [11]. Associations between mosquito exposure, malaria incidence and immune responses are further complicated by the fact that individuals with the highest malaria exposure will acquire protective immunity most rapidly and may experience lower malaria incidence in some settings [58], [59]. In general, it is complex to disentangle markers of exposure from markers of protection when analysing malaria blood stage antigens. Recent studies highlight the importance of considering malaria heterogeneity when determining the protective effect of antibody responses on clinical malaria. Initially, counterintuitive observations that higher blood stage immune responses were associated with increased malaria incidence [60], [61], were explained by adjusting for heterogeneity in malaria exposure and excluding non-parasitaemic individuals. This revealed a protective effect among immune responders, reflecting either true or surrogate humoral immune mediation [60]. This methodological challenge, first described by Bejon and colleagues [62], [63], has highlighted the need for markers that capture heterogeneity in malaria exposure but are not associated with clinical protection [58], [60], [61]. Markers of mosquito exposure, as described in this manuscript, may play this role by identifying those individuals most at risk of malaria.

No clear associations were apparent between *Anopheles* exposure at an individual level and antibody responses to any of the malaria-specific antigens (CSP, AMA-1, MSP-1, MSP-3, GLURP R1, GLURP R2). Anti-CSP reactivity might be expected to correlate with exposure to infected mosquito bites and therefore perhaps also with overall mosquito biting, but in our analysis did not. This may be a consequence of the relatively small sample size, and low EIR [11], [21]; in moderate to low endemic areas the proportion of infected vectors is frequently lower than 1% [15], [64], [65]. Contrary to this, individual-level anti-gSG6 responses were strongly associated with household *Anopheles* exposure. Interestingly, mosquito exposure was assessed towards the end of the study, starting approximately 15 months after the serum sample that was used for serology was collected. This suggests that heterogeneity in mosquito exposure is consistent over time in our study area, supporting the hypothesis of stable hotspots of malaria transmission [10], [22].

We previously showed that antibody responses to blood stage malaria antigens determined in clinic attendees reliably predicted spatial patterns in malaria incidence in a cohort of children living in the same area [11]. We here extended these analyses and showed that an individual’s antibody responses to MSP-1, MSP-3 and GLURP-R2 are all positively associated with subsequent malaria incidence. The selection of malaria antigens we used in this study was not intended to be exhaustive, nor did we aim to identify the malaria antigen with the highest discriminative power to detect variation in malaria exposure. We chose 4 malaria antigens to put our findings with gSG6 in an epidemiological context. Our findings are consistent with previous reports from areas of heterogeneous exposure where malaria specific antibody responses as markers of past exposure predict future exposure [60], [61]. Strikingly, in our analyses anti-gSG6 responses also provided a strong association with malaria incidence, indicating that malaria heterogeneity is associated with heterogeneous biting behaviour [12]. Unlike responses to transmission and blood stage malaria antigens [65], [66] responses to gSG6 confer no protection to malaria, thus avoiding any confounding associations with immunity and malaria incidence. In such a way, the gSG6 assay may provide a useful marker for exposure to malaria for use in clinical studies [58].

Though the sampling framework of the current study was not designed to evaluate the temporal dynamics of the anti-gSG6 response, there are indications that, as with responses to the salivary proteins of other haematophagous arthropods, it elicits short lived antibody responses, reflecting only recent *Anopheles* exposure [45], [46], [48]. As blood-feeding is transitory, and saliva is only released into the skin during probing with the majority likely to be re-ingested with the blood meal, this limits the development of a humoral immune response to mosquito saliva [67]–[69]. This short exposure to antigen explains the low anti-gSG6 responses observed among children from Korogwe. These low level responses highlight inherent problems in assessing exposure using an arbitrarily defined cut off for seropositive individuals. Identifying individuals never exposed to malaria is relatively straightforward but the same cannot be said for individuals never exposed to *Anopheles*, a genus which has a very wide geographical distribution. The nature of mosquito feeding, with the strength of the correlations observed in our analyses between spatial and individual level mosquito exposure and antibody OD, supports the use of antibody level rather than seroprevalence as a finer tool for assessment of *Anopheles* exposure intensity.

### Conclusions

This is the first report that antibody responses to the recombinant *An. gambiae* salivary protein gSG6 in children can reflect small-scale spatial variation in exposure to anophelines at village and household level. Importantly, our analysis also provides the first evidence for a reliable association between malaria incidence and anti-gSG6 response; a relationship only previously observed using whole *An. gambiae* saliva [44]. Caution is required in extrapolating findings from this study to other age groups because our analyses were restricted to plasma samples from children aged 9 months and a role of maternal transfer of IgG during breastfeeding can therefore not be excluded. This limitation of the current study does not alter our conclusions that these antibody responses are suitable markers of micro-epidemiological differences in *Anopheles* exposure. Potential uses for this assay include establishing *Anopheles* biting exposure to include indoor and outdoor biting, controlling for exposure in highly heterogeneous settings, and as a measure of receptivity to inform programs that are moving toward elimination where there is a high risk for re-introduction. However, its utility in low endemic and pre-elimination settings first needs to be assessed [8]. To this end, it will be important to establish the assays suitability for use with scalable antibody sources such as dried filter paper blood-spots. The identification and analysis of other salivary proteins may also help increase the sensitivity of the approach in such settings [70].
